# Microscopic and Biopharmaceutical Evaluation of Emulsion and Self-Emulsifying Oil with Cyclosporine

**DOI:** 10.3390/ph16121713

**Published:** 2023-12-11

**Authors:** Eliza Wolska, Małgorzata Sznitowska, Juliusz Chorążewicz, Katarzyna Krzemińska, Oliwia Szerkus, Aleksandra Radwańska, Michał J. Markuszewski, Roman Kaliszan, Krystyna Raczyńska

**Affiliations:** 1 Department of Pharmaceutical Technology, Medical University of Gdansk, Hallera 107, 80-416 Gdansk, Poland; msznito@gumed.edu.pl (M.S.);; 2 Department of Ophthalmology, Medical University of Gdansk, Smoluchowskiego 17, 80-214 Gdansk, Poland; 3 Department of Biopharmaceutics and Pharmacodynamics, Medical University of Gdansk, Hallera 107, 80-416 Gdansk, Poland

**Keywords:** emulsion, self-emulsifying oil (SEO), rabbit, Draize irritation test, cyclosporine, ophthalmic delivery

## Abstract

Among the currently available commercial eye drops with cyclosporine A (Cs) there is a lack of long-acting dosage forms and products with a concentration of the drug substance higher than 0.1%, although Cs is widely used in ophthalmology. The aim of the research was to conduct the microscopic and biopharmaceutical evaluation of two formulations, an emulsion (EM) and a self-emulsifying oil (SEO), both with 0.5% of Cs, proposed for use in eye drops, and the comparison of both. SEO eye drops with Cs or any other drug substance are currently not available as marketed products, and the highest concentration of Cs in the ocular emulsion is only 0.1%. The microscopic evaluation of the emulsion and the SEO after emulsification with water was carried out using a high-resolution digital microscopy. The properties of both preparations were compared using the high dynamic range function or optical shadow effect mode. Images in the 3D composition mode were also recorded. The *in vivo* study of the Cs formulations was performed on male albino rabbits. The eye tolerance of the preparations was assessed using the ocular irritation test, which is a modified Draize test. *Placebo* carriers (without the drug substance) were also subjected to irritation testing. The concentration of Cs in the tissues (cornea and conjunctiva) and fluids (tear fluid and aqueous humor) of the rabbit eye was determined after multiple instillations of Cs–EM or Cs–SEO. The tested preparations were compared using the digital microscopy technique, which highlights the features of the formulations and eliminates the risk of unnoticeable properties that are difficult to observe in classical optical microscopy. Both tested Cs-loaded formulations are classified as practically non-irritating. There were also no significant differences when testing the *placebo* carriers. After a topical administration, Cs was widely distributed in all tissues (e.g., in cornea 1.3 ng/mg and 1.0 ng/mg) and fluids of the eye (e.g., in tear fluid 11.6 µg/mL and 4.3 µg/mL), after the administration of Cs–SEO and Cs–EM, respectively. The obtained results allow us to recognize both tested formulations, the emulsion and the self-emulsifying oil with 0.5% Cs content, as carriers safe for ophthalmic use and effective in delivering the drug substance to the structures of the eye.

## 1. Introduction

Invariably, for years, most commercial preparations intended for topical application to the eye have been in the form of aqueous solutions [1,2,3]. It is a traditional dosage form, convenient for application via instillation and well tolerated by patients. On the other hand, this form is characterized by many disadvantages, such as short contact time with the surface of the eye, rapid leaching from the conjunctival sac, and the limited solubility or stability of the drug substance in the aqueous environment. Ultimately, all these aspects result in limited bioavailability (usually below 5%) [1,2,4].

Therefore, efforts are being made to develop new dosage forms for ophthalmic administration using modern carriers [5]. One of such carriers is emulsions already used in clinical practice [5]. Due to the aqueous dispersing phase, the oil-in-water emulsions are well tolerated [6,7]. At the same time, the dispersed oily phase makes it possible to dissolve the sparingly soluble drug substances. Another beneficial effect of using the emulsion is its stabilizing effect on the lipid layer of the tear film and, consequently, reduced water evaporation and the restoration of tear fluid integrity [8]. The approval by the registration agencies (FDA, EMA) of further preparations with drug substances in the form of oil-in-water emulsions for use in the treatment of eye diseases, as well as the introduction of “artificial tears” preparations in the form of emulsions, confirm the safety of their use [1,9].

Another innovative drug carrier, not yet available in the form of a commercial preparation, is self-emulsifying oil (SEO), which is a mixture of oil with a surfactant, sometimes also a co-surfactant or co-solvent [2,10]. Such composition, also called SEDDS (self-emulsifying drug delivery system), as a lipid-based formulation, is a promising carrier of substances that are slightly soluble in water [11,12]. SEO is spontaneously emulsified in contact with water and creates an emulsion *in situ*. As a result of such a mechanism, after application to the eye and due to the formation of an emulsion with tear fluid, SEO can avoid the disadvantages of the traditional oily solution (vision disorders, discomfort, burning). Moreover, the spontaneous formation of an emulsion upon dilution via tear secretion in the ocular surface advantageously presents the drug in a dissolved form and the small droplet size, which provides a large interfacial surface area for drug absorption [13]. SEOs are considered primarily as carriers that extend the time of contact with the surface of the eye in order to increase bioavailability [2,4]. An important advantage of SEO as a dosage form is the presence of the active substance in the solution form, hence avoiding the dissolution step required, for example after the application of a suspension [4]. Due to good solubility in SEO, it becomes possible to administer a preparation with a higher concentration of the active substance, less frequently instilled (limiting systemic side effects after nasolacrimal duct absorption) [2]. The increased solubility of active substances, prolonged pre-ocular retention, and enhanced drug absorption are particularly desirable features of the dosage form in ophthalmic therapy [4]. Moreover, SEO is considered as a carrier of substances that are not only insoluble in water but also unstable in water [14].

This study employed Cs as a model drug. This selection was based on the fact that Cs is widely used in the ophthalmic therapy of various eye diseases, mainly as an anti-inflammatory treatment. Although Cs is widely used, it remains a challenge to administer it with conventional topical ophthalmic delivery systems and with satisfactory bioavailability, because Cs has a large molecular weight and hydrophobicity, resulting in poor aqueous solubility [15]. Although since 2002 international registration agencies (FDA and EMA) have approved several Cs preparations for use in the eye, almost all of them are in the form of emulsion in which the concentration of the active substance does not exceed 1 mg/mL [1]. Meanwhile, the concentration of Cs in eye drops used in medicine is 0.5%, 1%, or even 2%. The only available solution in such a situation is the use of compounded oily eye drops prepared in retail and hospital pharmacies [16].

In this study, we focused on the microscopic and *in vivo* evaluation of two formulations, emulsion and SEO, both containing 0.5% Cs, in the form of eye drops. The *in vivo* studies, namely ocular irritation test via modified Draize test and biopharmaceutical evaluation, were investigated in rabbits as animal models to assess the application potential and the safety of use of the tested formulations. The developed preparations were applied to the eye to determine the level of Cs in individual tissues of the eyeball assessed after multiple ocular administrations. A comparative microscopic evaluation of both formulations was also carried out using a modern imaging technique. According to our best knowledge, SEO and emulsion were evaluated for the first time using a high-resolution digital microscope.

## 2. Results

### 2.1. Microscopic Characterization of Emulsion and SEO

**Figure 1:**
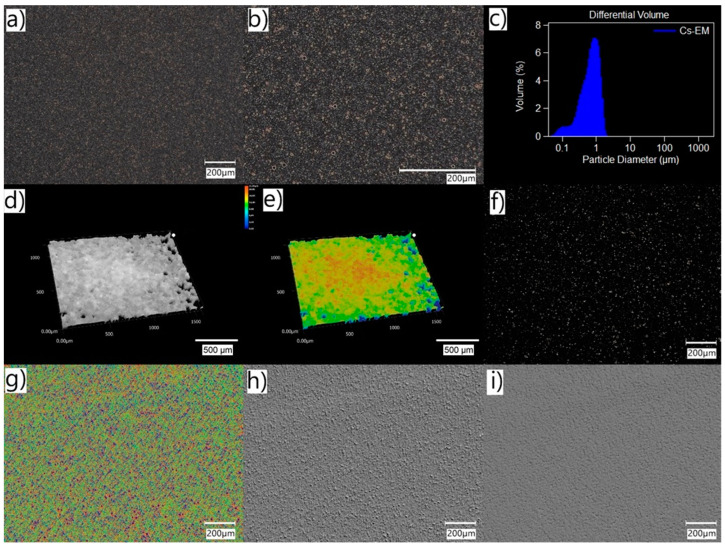
Digital microscope images of Cs–EM formulation obtained with HDR function: (**a**,**b**,**f**); 3D mode: (**d**,**e**); and optical shadow effect: (**g**–**i**). Section: (**c**) presents the particle size distribution in the tested emulsion obtained via laser diffraction (point 4.3).

Figure 1 shows the structure of the tested o/w emulsion (Cs–EM) with the use of various high-resolution digital microscope options, described in point 4.4. The examined emulsion is homogeneous in the microscopic image. No agglomerates or any particles/precipitate of the active substance dissolved in the oil phase are visible. Due to their size (generally below 5 µm), emulsion droplets (droplets of oil dispersed in the aqueous phase) appear as dots or circular rings. The observation of oil droplets with a size of about 1 μm is possible in HDR mode, which increases the level of detail seen in the image via increasing the color gradation, as can be seen in Figure 1a,b. This function captures multiple frames of the image with different degrees of brightness, adjusting the shutter speed (these are further composed into a single high-resolution image). Depending on the microscope settings, images with higher contrast (Figure 1f) or images with 3D structures (Figure 1d,e) were also obtained. In the case of emulsions, images in which the spatial structure of small drops was most visible are the images made in the optical shadow mode. Regardless of the microscope functions used, all the obtained images (Figure 1) confirm the size of the emulsion droplets measured via laser diffraction (Figure 1c). In Cs–EM, the average droplet size was 0.73 µm and 90% of the particles were below 1.23 µm.

**Figure 2:**
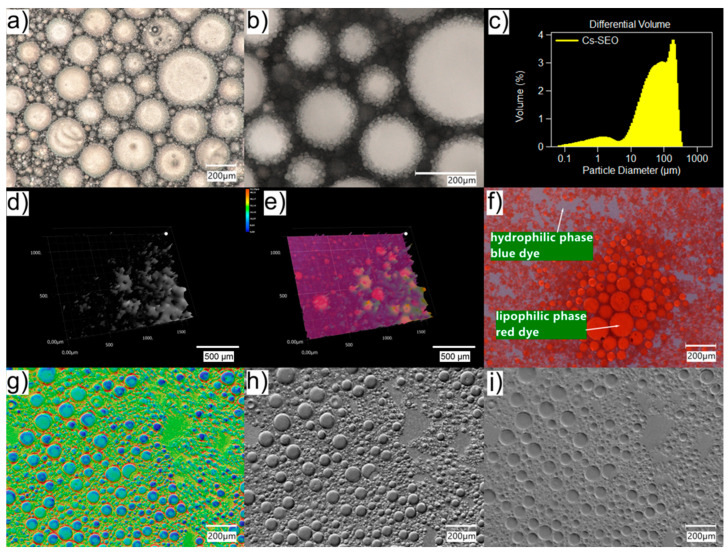
Digital microscope images of Cs–SEO formulation obtained with HDR function: (**a**,**b**); 3D mode: (**d**,**e**); after staining the water and oil phases with hydrophilic and lipophilic dyes, respectively: (**f**); and optical shadow effect mode: (**g**–**i**). Section: (**c**) presents the particle size distribution in the tested SEO obtained via laser diffraction (point 4.3).

**Figure 3:**
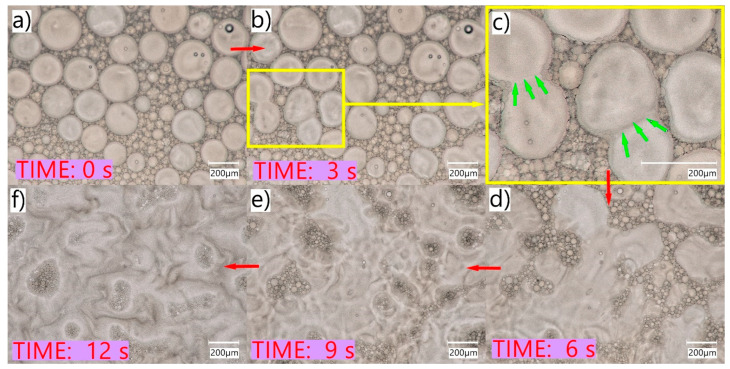
Digital microscope images of the Cs–SEO coalescence process. Green arrows indicate selected places where the oil droplets begin to coalesce and phase separation disappears. Red arrows indicate the progress of changes occurring during coalescence—the direction of reading is from (**a**–**f**).

Figure 2 shows Cs–SEO after emulsification with water. Unlike Cs–EM, the droplets of the dispersed phase in the obtained o/w emulsion had a fairly large size and were in a very wide range of sizes (Figure 2a). This fact is confirmed by the results of the particle size study carried out using laser diffraction (Figure 2c). The mean Cs–SEO particle size after emulsification was 84.3 µm (90% of droplets were below 199.0 µm). In the microscopic image, large oil droplets surrounded by numerous, very small droplets are clearly visible (Figure 2b). Images made in the optical shadow mode (Figure 2g–i), also in the color version, capture the size diversity and spatial nature of the droplets very well, which otherwise might go unnoticed. A similar effect can also be observed in the 3D images. The 3D mode provides a powerful observation of the tested, multiparticulate formulation from various directions, which is difficult to perform in a 2D image. The nature of the obtained emulsion (o/w) was confirmed with the addition of dyes methylene blue and Sudan III, soluble in the hydrophilic and lipophilic phases, respectively. The image of the emulsified and stained Cs–SEO with red oil droplets and blue dispersing water phase is shown in Figure 2f. As in the Cs–EM studies, no Cs particles or precipitate were visible. The obtained system, however, was characterized by significant heterogeneity and dynamics: the drops of emulsion spontaneously changed their sizes. In addition, after a short time of observation (1–2 min) of the sample without the coverslip, the coalescence effect was observed, which is the result of the evaporation of water from the sample. This phenomenon is shown in Figure 3.

In the microscopic image, the emulsion drops were observed to merge with each other during their movement. The drops approached each other and then merged into one larger drop (the effect of coalescence). This phenomenon concerned primarily larger drops. The small drops that had previously surrounded the larger drops, after they merged, formed clusters of droplets in SEO (Figure 3). The entire described process was quite fast, as shown in Figure 3.

### 2.2. In Vivo Eye Irritation Test

The summary of the irritation test results is shown in Table 1. None of the tested formulations administered to the rabbit eye caused any changes in such eye structures as the cornea or the iris. Slight differences were observed only when evaluating the conjunctiva, although, also in this case, the scores grading the severity of ocular lesions were almost zero (Table 1).

The changes observed during this study, such as redness or swelling of the conjunctiva, appeared slightly more often after SEO than after EM application, regardless of whether it was just a *placebo* carrier or a Cs-loaded dosage form. The number of rabbits that showed a positive response to Cs–EM is not more than three out of eight rabbits (Table 1), which means that was slightly less than after the administration of Cs–SEO (four out of eight rabbits). Meanwhile, the application of the *placebo* emulsion caused a reaction in only one eye in one rabbit. Despite these subtle differences, guided by the ranges presented in point 4.5.1, EM should be considered as a nonirritating carrier, and all the other tested formulations, i.e., Cs–EM, SEO, and Cs–SEO, as practically nonirritating.

Based on the collected results, it can therefore be concluded that the tested emulsions and SEO formulations produced a negligible irritation to rabbit eyes. However, also important is the fact that any observed signs of irritation have resolved before the next application. Taking into account also the visual observations and assessment of rabbits’ behavior carried out during and immediately after the application of all tested formulations, it can be concluded that the tested formulations did not produce any significant discomfort to rabbit eyes.

**Figure 4:**
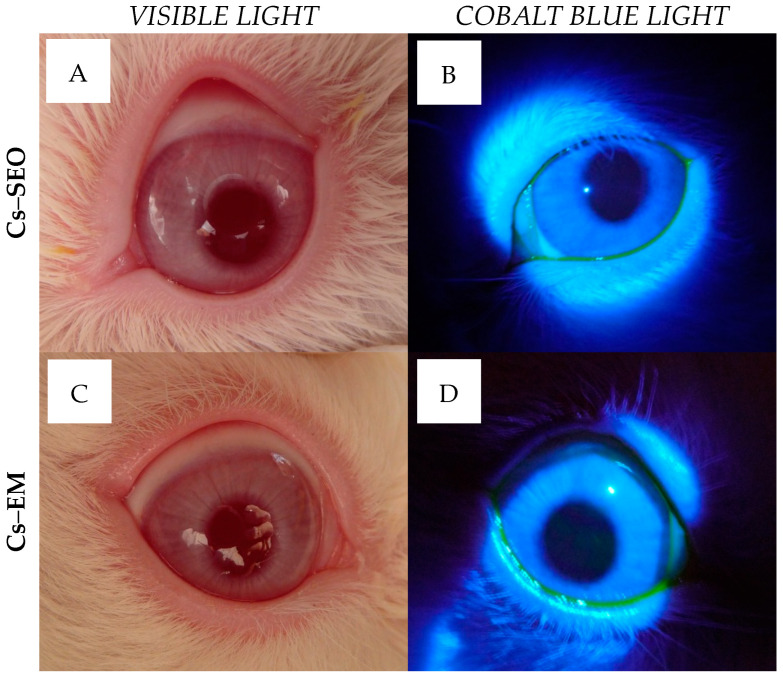
Local ocular reaction observed under the slit lamp after *in vivo* instillation of SEO with 0.5% Cs (**A**,**B**) or emulsion with 0.5% Cs (**C**,**D**) for 5 days. Pictures were taken in visible light (**A**,**C**) or in Cobalt blue light after fluorescein instillation (**B**,**D**).

When the eyes were examined with an ophthalmoscope, after the instillation of sodium fluorescein, no signs of damage or breaks in the continuity of the epithelium in the cornea were detected. The corneas were clear and the ocular surface remained intact, which is visible in Figure 4. No other changes were seen in the tested eyes (Figure 4) or the behavior of the rabbits accompanying the administration of the *placebo* and Cs-loaded formulations.

### 2.3. In Vivo Ocular Distribution of Cs

**Figure 5:**
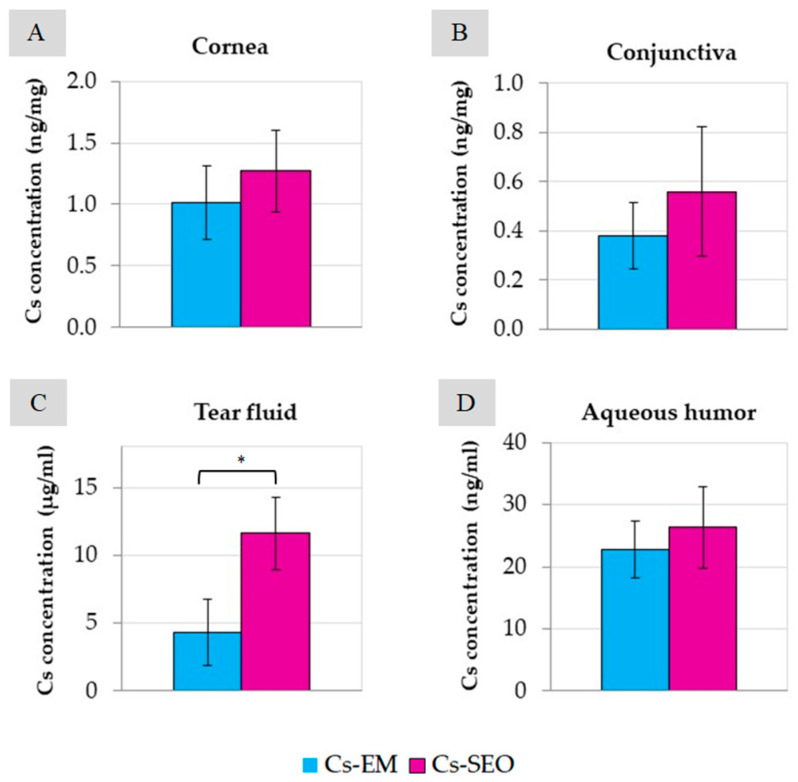
Biodistribution (*n* = 8, mean ± SD, *—statistically significant difference) of Cs in rabbit eyes after multiple (7 days, 14 instillations) topical administrations of Cs-loaded (0.5%) emulsion and SEO: (**A**) cornea, (**B**) conjunctiva, (**C**) tear fluid, (**D**) aqueous humor.

The *in vivo* distribution of Cs among ocular tissues in the rabbit eye was examined after a 7-day administration of Cs–EM and Cs–SEO. As described in Section 4.5.2, both formulations containing 0.5% of Cs were administered twice a day. With a single administration of 20 µL of the Cs-loaded preparation, the single dose of Cs was approximately 100 µg. The concentration of Cs in different rabbit eye tissues after the administration of the emulsion and SEO is shown in Figure 5.

The highest Cs concentration was obtained in the tear fluid (Figure 5C), and it was approximately 4.3 µg/mL and 11.6 µg/mL after the application of the emulsion and SEO, respectively. Also, only in the case of tear fluid, a statistically significant difference in concentration between the tested preparations was found.

The concentration of Cs in the remaining tissues and aqueous humor showed no significant differences between Cs–SEO and Cs–EM (*p* < 0.05). The determined concentrations of Cs in the cornea (Figure 5A) were very similar in both tested formulations (1.0 ng/mg in EM and 1.3 ng/mg in SEO). The lowest concentration of Cs (less than 0.6 ng/mg) was found in the conjunctiva (Figure 5B), regardless of the drug carrier used. Concentrations obtained in the aqueous humor (Figure 5D) of the eyeball were at a relatively high level (approximately 23–26 ng/mL).

## 3. Discussion

Ophthalmic emulsions are a well-known and already used dosage form, while, thanks to ongoing research [2,13,14], the self-emulsifying oils have the potential to become an alternative drug carrier for use in ophthalmology. The simple composition and uncomplicated method of preparation, as well as the ingredients already being used in eye medications (oils, non-ionic surfactants), create a chance to introduce SEO to widespread use. For the above reasons, good SEO tolerance is expected after application to the eye, also due to the presence of a surfactant in the composition. Well known, although not widely used, are oily eye drops. Their limited use is mainly due to the discomfort caused to the patient after application (burning sensation, irritation, blurred vision [17,18,19,20,21]). The purpose of using a surface-active substance in SEO is to ensure quick and effective emulsification in contact with water, and, in the case of application to the eye, easier mixing of SEO drops with tear fluid, consequently also reducing/eliminating discomfort after the application. The surfactant can also favorably affect the solubility of the drug substance (both in the presence and absence of water). Therefore, SEO is a dosage form that could be a good carrier for substances sparingly soluble in water, which are difficult to administer in the form of an aqueous carrier.

In the conducted experiments, SEO and o/w emulsion were tested, for the production of which castor oil was used. The optimal composition of the currently investigated SEO was selected on the basis of previous research [22]. The proposed self-emulsifying oil with Cs for ophthalmic application is characterized by a transparent appearance compared to a milky-colored o/w emulsion. In both carriers, the drug substance (Cs) was completely dissolved in oil at a concentration of 0.5%. Castor oil was selected based on its physicochemical properties and traditional use in eye medications, including eye drops with Cs compounded in hospital or retail pharmacies [16,18,23]. The concentration (20%) of the oily phase in the emulsion was selected based on our previous studies as well as the composition of the extremely stable 10% or 20% parenteral emulsions (marketed products) commonly used in intravenously-administered parenteral nutrition mixtures.

Tween 80 (polysorbate 80) was used as a surfactant in both tested formulations, due to being considered non-toxic and non-irritating as a non-ionic surface-active compound. It is commonly used not only in drug formulations (including eye drops) but also in cosmetics and food products [24,25]. Our previous studies also confirmed the good tolerance of the ophthalmic dosage forms (SLM dispersion in the form of eye drops) with polysorbate at a concentration of 3% by the rabbit eye [26]. The advantage of polysorbate is not only increasing the solubility of sparingly soluble substances but also the ability to improve their bioavailability [25].

When considering the bioavailability of a drug substance after administration to the conjunctival sac, the residence time of the dosage form in contact with the eye surface and the release of the active substance from the drug carrier should be taken into account. In the case of SEO, the mechanism of emulsification with the tear fluid should also be considered, although there is little data in this regard.

Therefore, valuable additional information about the properties like emulsification and the stability of the system can be gained using the light microscopical inspection of the size, structure, and behavior of emulsion droplets. Emulsions o/w are quite difficult to observe in a traditional optical microscope due to the small size of oil droplets (usually below 1 µm). In addition, the high transparency of both the water and oil phases reduces their contrast against the background. The use of a digital microscope with an optical system that combines high resolution with a large depth of field allowed for the better visualization of small emulsion droplets (Figure 1). Moreover, using the HDR function, it was possible to obtain an image of the emulsion drops with higher color gradation. The use of different lighting conditions was also helpful in the imaging (also in the 3D model) of drops in the emulsified SEO (Figure 2).

It is known that the ophthalmic oily solutions used in clinical practice are less tolerated by patients than the aqueous formulations. On the other hand, the oily solution is removed from the conjunctival sac more slowly than the aqueous solution. Due to the presence of the surfactant, SEO can be emulsified and re-emulsified reversibly, mainly due to better (than in the case of oil) miscibility of SEO with tear fluid. As a result, better tolerance by the eye with prolonged contact with the eyeball is expected. As observed, the tested SEO very easily emulsifies with water, either after mixing in an Eppendorf tube for a few seconds on a vortex mixer or by hand. This gives grounds to assume that a similar process will take place on the surface of the eyeball after SEO application, as a result of mixing with tear fluid during blinking.

As observed in non-coverglass microscopy studies, the evaporation of the water and the decreasing volume of the aqueous phase resulted in the reversal of the emulsification process, whereby larger oil droplets coalesced to form a continuous oily phase (Figure 3). Such a phenomenon may occur in the case of SEO eye applications with a small volume of tear fluid (e.g., patients with dry eye syndrome), in which the SEO sensation may be similar to oil application, due to the limited amount of water phase for emulsifying the instilled preparation. This is important from the point of view of the patient who may experience discomfort after using eye drops and should be taken into account by a physician when selecting the dosage form appropriate for a particular patient. At the same time, the quick and easy emulsification of SEO in patients’ eyes without significant disturbances in the amount of tear fluid may affect the amount of dissolved active substance present in the tear fluid and, consequently, also its bioavailability. This effect was observed in a study on rabbits, in which a significant difference between Cs–EM and Cs–SEO and the highest concentrations of the drug substance were observed in the tear fluid (Figure 5).

Tested formulations were very well tolerated in rabbits. During the irritation study, there were no changes in cornea or iris observed after the administration of *placebo* and Cs-loaded formulations. Some slight macroscopic signs of eye irritation were observed only in conjunctiva, as shown in Table 1. Admittedly, based on the analysis of the data collected in Table 1, some subtle differences could be found: firstly, between *placebo* carriers and Cs preparations; and secondly, between Cs–EM and Cs–SEO. However, there is no basis for a clear distinction between the tolerance of Cs-loaded emulsion and Cs–SEO after the administration to the rabbit eye, taking into account a very low degree of conjunctiva irritation and low average score value (Table 1). Furthermore, the *in vivo* ophthalmoscopy examination did not reveal any pathological symptoms in the eye (Figure 4), and the rabbits, both during and immediately after application, did not show any macroscopic symptoms or behaviors that could indicate discomfort, regardless of the tested formulation. As recommended, to avoid being misled by atypical outliers, the ratio of eyes with at least a threshold reaction to all tested eyes should be recorded in each test group. The recognition of positive responses in a particular group is justified if symptoms are observed in more than half of the tested rabbits [27]. In our studies, the number of positive responses was always observed in less than half of the tested eyes (Table 1), regardless of the stage of the study (*placebo* or Cs-loaded carriers) or the tested formulation (both emulsion and SEO). Moreover, rabbit eyes are generally recognized as being more susceptible to irritating substances than human eyes [27,28]; therefore, the presented results of irritating study allow to expect that SEO will be as well accepted by the human eye as by the rabbit eye. Thus, on the basis of the obtained results and in relation to the adopted classification, the tested preparations should be classified as practically non-irritating. Summarizing, the obtained results allow us to recognize SEO and EM with 0.5% of Cs as safe formulations for ophthalmic use.

The therapeutic level of Cs required to weaken the immune response and inflammation in eye tissues is 0.05–0.3 ng/mg of tissue [29,30]. In our study, in the tested tissues (cornea and conjunctiva, Figure 5), the concentration of Cs was even 25 times higher than the therapeutic level, both after the use of the emulsion and SEO. The Cs concentration in the aqueous humor was slightly below this range (approximately 0.02 ng/µL). However, there are reports that after the administration of the commercially available Restasis emulsion, the concentration of Cs was many times lower (<1 ng/mL) [20]. The highest concentration of Cs was determined in the tear fluid (Figure 5C), in which a significant difference was also found between the emulsion and the SEO. In our study, Cs concentration in ocular tissues and fluids was measured only at one time point (3 h after the last application). After SEO administration, the Cs concentration was almost three times higher (about 11.6 µg/mL) than after emulsion instillation (about 4.3 µg/mL) for the same period of time (7 days). A clear difference (statistically significant) in the concentration of Cs determined in the tear fluid suggests longer residence time of the SEO than of the emulsion in the conjunctival sac. Such a difference may be due to the different behavior of the emulsion and the self-emulsifying oil in the conjunctival sac. Due to the presence of a surfactant, SEO may result in a higher concentration of Cs on the surface of the eyeball via various mechanisms (the formation of micelles and the solubilization of sparingly soluble Cs, easier emulsification with tear fluid). This effect, combined with the longer time when the SEO stays in contact with the eye surface, should be considered beneficial. Therefore, due to the sustained release of the drug substance from the SEO dosage form and prolonged contact with the surface of the eye, better effectiveness can be expected.

To summarize, the justification for conducting the presented research is the significant frequency of Cs use in the treatment of eye diseases, with a simultaneous low diversity of available commercial products (both in terms of dosage form and Cs concentration). The obtained *in vivo* results confirm the good tolerance of both proposed carriers (emulsion and SEO) and preparations with the drug substance (Cs–EM, Cs–SEO) by the rabbit eye. It was expected based on the composition of the oily solutions used in ophthalmology and the properties of the emulsion already available in medicine. Not only good tolerance, but above all, the effectiveness in ensuring therapeutic concentrations of Cs in individual eye tissues, confirms the advisability of developing a new long-acting, easy to produce dosage form not previously used in medicine, i.e., the self-emulsifying oil. The use of high-resolution digital microscopy imaging allowed for not only an innovative presentation of the properties of the tested formulations, but also for the consideration of processes that could occur on the surface of the eyeball in contact with the tear fluid and under the influence of blinking and their implications for the patient. The lack of influence of the active substance additive on the SEO properties allows us to expect that the proposed self-emulsifying oil could also be a suitable carrier for drug substances other than Cs. The usefulness or limitation of its use will certainly be determined by the solubility of the drug substance in SEO. Finally, the advisability of developing a dosage form of Cs emulsion already used in medicine was also demonstrated, but in a higher, currently unavailable, concentration of 0.5%.

## 4. Materials and Methods

### 4.1. Materials

Cyclosporine A (Cs) was obtained from LC Laboratories (Boston, MA, USA) and cyclosporine D (CsD), which was used as an internal standard, was purchased from Santa Cruz Biotechnology (Santa Cruz, CA, USA). Tween 80 (polysorbate 80) was purchased from Sigma–Aldrich (St. Louis, MO, USA) and castor oil from Sigma–Aldrich (Seelze, Germany); methanol and acetonitrile were purchased from Merck (Darmstadt, Germany). All other chemicals used were of analytical reagent grade. A high-quality water was obtained from a Milli-Q system (Millipore, Milford, MA, USA).

### 4.2. Preparation of Tested Formulations

Composition of the emulsion and SEO with Cs is presented in Table 2. Both formulations contained 0.5% of dissolved Cs as the drug substance. The emulsion with castor oil (EM) was prepared by employing a hot-stage high-pressure homogenization (High Pressure Homogenizer APV-2000, APV Gaulin, Lopik, the Netherlands). Cs–EM was obtained by dissolving the active substance in the ready-made emulsion on a magnetic stirrer. The emulsions were thermally sterilized in an autoclave.

SEO was prepared by mixing the surfactant (Tween 80) with castor oil on a magnetic stirrer. Then Cs was added and stirring was continued, with slight heating, until complete dissolution of drug substance (Cs–SEO). SEO formulations were sterilized via filtration (0.2 µm) under aseptic conditions. *Placebo* preparations without active substance (indicated in Table 2 as EM and SEO) were prepared for comparative studies.

### 4.3. Droplet Size Measurement

The droplet size distribution was measured via laser diffraction technique in a device (Beckman Coulter LS 13 320, Indianapolis, IN, USA) equipped with the PIDS function (Polarization Intensity Differential Scattering). A Universal Liquid Module (ULM) was used to measure the emulsion directly and the SEO after prior mixing and emulsification with water, in a 1:1 ratio. The tested formulation was added to the measuring cell until the level of obscuration parameter, recommended by the apparatus, was reached.

### 4.4. Microscopic Evaluation

Microscopic analysis of the emulsion and SEO was performed using a digital microscope (Keyence VHX 7000, Keyence International, Mechelen, Belgium) equipped with a high-performance zoom lens, Z20T (the magnification range: 20×–200×).

During the observation, different visualization models were used: HDR function (the high dynamic range function), optical shadow effect mode (OSEM), live depth composition, or 3D composition (which can be displayed in monochrome or height/color). The implemented software tool was used for image analysis.

The tested formulations, Cs–EM and Cs–SEO (emulsified with water 1:1), were compared in terms of the size and uniformity of the oil phase drops, or the precipitation of the active substance, and changes in Cs–SEO were observed due to the loss of water from the system. The Cs–SEO formulation was also observed after staining the phases with the following dyes: lipophilic Sudan III and water-soluble methylene blue.

### 4.5. In Vivo Studies in Rabbits

This study on rabbits was conducted after obtaining the approval from the Independent Bioethics Committee on Scientific Research of the Medical University of Gdansk (41/2012, LKE50/2013). Blanc de Termonde rabbits (albino males), weighing 2.5–3.5 kg, were used in the studies. Rabbits were supplied by an accredited supplier (Laboratory Animals Breeding, Nieborow, Poland). All the animals were healthy and free of clinically observable abnormalities. Prior to this study, the rabbits were acclimatized under standard conditions for 7 days. They were individually housed in cages and maintained in night and day cycles, with free access to standard diet and water.

#### 4.5.1. *In Vivo* Eye Irritation Test

The *in vivo* evaluation was conducted following the low-volume eye test procedure, which is a modification of the Draize test [27,31,32]. At each stage, before the administration of tested formulations, eyes of the rabbits were inspected to make sure that they were free of irritation, defects, or damage. This study was conducted in two stages: the first with *placebo* carriers and the second (after a break of two weeks) with Cs-loaded emulsion and SEO. The condition for conducting the second stage was the number of points obtained in the first step below 40 (Table 3). Each tested formulation was instilled into the 8 eyes in a volume of 10 µL (*placebo* carriers) or 20 µL (Cs-loaded emulsion and SEO) as eye drops, directly onto the corneal surface. Other 8 eyes were treated with the same volume of normal saline (0.9% NaCl) as the control. The preparations were administered crosswise (to the right or left eye), and the sodium chloride solution was administered to the other eye.

When *placebo* emulsion and SEO were tested, they were administered three times at intervals of 8–10 h. The condition of the ocular tissues (conjunctiva, iris and cornea) was assessed approximately 30 min after each instillation (A.1.–A.3.), as well as 12, 24, and 48 h after the last instillation (B.12 h–B.48h). Eye examination was performed using a standard ophthalmoscope (Beta 200 S, Heine Optotechnik, Herrsching, Germany). Preparations with Cs were administered for 7 days, two times a day. The condition of ocular tissues was monitored approximately 30 min after instillation, once a day.

As previously described [26], according to the Draize eye test, the total irritation score for one eye is represented by the sum of the irritation scores for cornea, iris, and conjunctiva (as shown in Table 3). The mean values from eight treated eyes, at each time point, were calculated for each *placebo* carrier and Cs-loaded formulation. Based on the results, the applicable scale called the maximum average score (MAS) can reach 110 points (Table 3) [27]. According to the classification by Kay and Calandra [32,33], the evaluation criteria are as follows: nonirritating (0 ≤ MAS < 0.5), practically nonirritating (0.5 ≤ MAS < 2.5), minimally irritating (2.5 ≤ MAS < 15), mildly irritating (15 ≤ MAS < 25), moderately irritating (25 ≤ MAS < 50), severely irritating (50 ≤ MAS < 80), extremely irritating (80 ≤ MAS < 100), and maximally irritating (100 ≤ MAS < 110).

In both stages, in order to better inspect the rabbit eyes, sodium fluorescein solution (0.5% *w*/*w*) was instilled into the eye on the fifth day (6 h after the morning administration of Cs-loaded formulations), or after completion of administration of the *placebo* carriers. The fluorescein allowed for the selective visualization of the potential corneal damage, which could be observed using a slit lamp equipped with a blue filter.

#### 4.5.2. *In Vivo* Ocular Distribution of Cs

*In vivo* distribution of drug substance in the rabbit eye tissues was studied after 7-day administration (twice a day) of Cs-loaded emulsion or SEO. The distribution study was conducted in parallel with the second stage of the irritation study (the same drug administration regimen).

As previously described [26], at 3 h following the last instillation of the formulations, ocular tear samples were collected using a Schirmer strip (Biotech Vision Care, Khatray, India). The animals were sacrificed via an overdose of sodium pentobarbital. After that, the eye tissues and fluid (conjunctiva, cornea and aqueous humor) were immediately isolated for analysis, as described in previous studies [26].

The concentration of Cs in all the tested tissues and fluids separated from the eyes of rabbits was determined with UHPLC method [34]. Cyclosporine D was used as an internal standard. The extraction method and procedures of sample preparation before UHPLC examination, as well as UHPLC analysis, were the same as those reported in previous studies [34]. Chromatographic analysis was performed with an Agilent 1290 Infinity system (Palo Alto, CA, USA), composed of binary high-pressure pump (G4220B), autosampler (G4226A), diode array detector (G4212A DAD), and a thermostated column compartment (G1316C). Chromatographic separation was performed on XB-C18 Kinetex column (Phenomenex, Torrance, CA, USA) thermostated at temperature of 60 °C. The chromatography was performed by applying a step gradient elution mode with the mobile phase flow rate 0.5 mL/min and the injection volume 10 µL. The used method was validated according to the ICH Guidelines (ICH EMEA, 2006). The developed assay was specific, sensitive (LOD = 6 ng/mL and LOQ = 18 ng/mL), and linear within the analytes concentration range of 0.018–5 µg/mL, with the correlation coefficient of 0.999. The above and remaining analysis and validation conditions have been previously described in detail [34].

### 4.6. Statistical Analysis

The statistical analyses were conducted using Statistica software (StatSoft, Ver. 13.3, TIBCO Software Inc., Palo Alto, CA, USA). The statistical significance of differences was tested using a one-way analysis of variance (ANOVA). Differences were considered to be significant at level of *p* < 0.05.

## 5. Conclusions

The comparative microscopic assessment of both tested formulations, SEO and emulsion, was performed for the first time using a high-resolution digital microscope. The microscopic evaluation using advanced imaging techniques allowed not only to compare the tested formulations (EM and SEO) on both flat (2D) and three-dimensional (3D) images, but also to predict the course of phenomena (e.g., emulsification and coalescence) occurring in the eye after SEO instillation. The emulsification of SEO with the tear fluid, also accelerated with eye blinking, may have consequences after SEO application in humans, who may not experience as much discomfort as after the administration of an oily solution.

The obtained results of eye irritation test allow us to recognize both tested formulations, the emulsion and the self-emulsifying oil (SEO) with 0.5% Cs content, as safe formulations for ophthalmic use. When testing castor oil formulations containing the drug substance (Cs–EM and Cs–SEO), very similar results were obtained to those of the same *placebo* carriers (EM and SEO).

After the local administration of the tested formulations, o/w emulsion and SEO, to the rabbit eye, it can be concluded that Cs was widely distributed in the ocular tissues and fluids, achieving therapeutic concentrations.

The developed Cs–SEO formulation is a promising alternative to conventional eye drops due to good eye tolerance, similar or better drug distribution in ocular tissues and fluids via its longer precorneal residence time, easy emulsification with tear fluid, and the ability of prolonged drug release.

## Figures and Tables

**Table 1:** *In vivo* conjunctiva irritation results of the *placebo* and Cs-loaded formulations, according to the Draize eye irritation test (*n* = 8).

Formulation (Concentration of Cs %)	N^o^ of Observation	N^o^ of Eye = 8	Eyes with Symptoms/without Signs of Irritation	Average Score ± SD
	A.1.	0/0/0/0/ **2** /0/0/0	1/7	0.25 ± 0.7071
	A.2.	0/0/0/0/0/0/0/0	0/8	0.0
**EM**	A.3.	0/0/0/0/0/0/0/0	0/8	0.0
**(placebo)**	B.12 h	0/0/0/0/0/0/0/0	0/8	0.0
	B.24 h	0/0/0/0/0/0/0/0	0/8	0.0
	B.48 h	0/0/0/0/0/0/0/0	0/8	0.0
	1.	0/0/0/0/0/0/0/0	0/8	0.0
	2.	0/0/0/0/0/0/0/0	0/8	0.0
	3.	0/0/0/0/ **2** /0/0/ **2**	2/6	0.50 ± 0.9
**Cs-EM**	4.	0/0/0/0/0/0/0/0	0/8	0.0
**(0.5% Cs)**	5.	0/0/0/0/0/0/0/0	0/8	0.0
	6.	0/0/0/0/0/0/ **2** /0	1/7	0.25 ± 0.7
	7.	0/0/0/0/0/0/0/0	0/8	0.0
	8.	0/0/0/0/0/0/0/0	0/8	0.0
	A.1.	0/ **4** /0/0/0/0/ **2** /0	2/6	0.75 ± 1.4880
	A.2.	**4** / **2** /0/0/0/0/0/0	2/6	0.75 ± 1.4880
**SEO**	A.3.	**4** /0/0/0/0/0/0/0	1/7	0.50 ± 1.4142
**(placebo)**	B.12 h	0/0/0/0/0/0/0/0	0/8	0.0
	B.24 h	0/0/0/0/0/0/0/0	0/8	0.0
	B.48 h	0/0/0/0/0/0/0/0	0/8	0.0
	1.	0/0/0/0/0/0/0/0	0/8	0.0
	2.	0/0/0/ **2** /0/0/0/0	1/7	0.25 ± 0.7071
	3.	0/0/0/0/0/ **2** /0/0	1/7	0.25 ± 0.7071
**Cs-SEO**	4.	0/0/0/0/0/0/0/ **2**	1/7	0.25 ± 0.7071
**(0.5% Cs)**	5.	0/0/0/0/0/0/0/0	0/8	0.0
	6.	0/0/0/ **4** /0/0/0/0	1/7	0.50 ± 1.4142
	7.	0/0/0/ **2** /0/ **4** /0/ **4**	3/5	1.25 ± 1.8322
	8.	0/0/ **2** / **2** /0/0/0/0	2/6	0.50 ± 0.9258
	1.	0/0/0/0/0/0/0/0	0/8	0.0
	2.	0/0/0/0/0/0/0/0	0/8	0.0
	3.	0/0/0/0/0/0/0/0	0/8	0.0
**0.9% NaCl**	4.	0/0/0/0/0/0/0/0	0/8	0.0
**(–)**	5.	0/0/0/0/0/0/0/0	0/8	0.0
**Control**	6.	0/0/0/0/0/0/0/0	0/8	0.0
	7.	0/0/0/0/0/0/0/0	0/8	0.0
	8.	0/0/0/0/0/0/0/0	0/8	0.0

**Table 2:** The composition (*w*/*w* %) of the investigated ophthalmic formulations.

Formulation	Cs	Castor Oil	Tween 80	Glycerol	Water
SEO	-	95.0	5.0	-	-
Cs–SEO	0.5	94.5	5.0	-	-
EM	-	20.0	3.0	1.8	75.2
Cs–EM	0.5	20.0	3.0	1.8	74.7

Cs: cyclosporine A, SEO: self-emulsifying oil, EM: o/w emulsion.

**Table 3:** The Draize scale for grading the severity of ocular lesions [27,32].

Eye Tissue	Scale	Maximum
Cornea	opacity (1–4) × area (1–4) × 5	80
Iris	grading value (1–2) × 5	10
Conjunctiva	[redness (1–3) + chemosis (1–4) + discharge (1–3)] × 2	20
**Total score**		**110**

If cornea, iris, or conjunctiva are normal, a score of 0 is assigned to each parameter.

## Data Availability

Data are contained within the article.

## References

[1] Mazet R., Yaméogo J.B.G., Wouessidjewe D., Choisnard L., Gèze A. (2020). Recent Advances in the Design of Topical Ophthalmic Delivery Systems in the Treatment of Ocular Surface Inflammation and Their Biopharmaceutical Evaluation. Pharmaceutics.

[2] Elbahwy I.A., Lupo N., Ibrahim H.M., Ismael H.R., Kasem A.A., Caliskan C., Matuszczak B., Bernkop-Schnürch A. (2018). Mucoadhesive self-emulsifying delivery systems for ocular administration of econazole. Int. J. Pharm..

[3] Ahmed S., Amin M.M., Sayed S. (2023). Ocular Drug Delivery: A Comprehensive Review. AAPS PharmSciTech.

[4] ElKasabgy N.A. (2014). Ocular supersaturated self-nanoemulsifying drug delivery systems (S-SNEDDS) to enhance econazole nitrate bioavailability. Int. J. Pharm..

[5] Souto E.B., Dias-Ferreira J., López-Machado A., Ettcheto M., Cano A., Espuny A.C., Espina M., Garcia M.L., Sánchez-López E. (2019). Advanced formulation approaches for ocular drug delivery: State-of-the-art and recent patents. Pharmaceutics.

[6] Lallemand F., Daull P., Benita S., Buggage R., Garrigue J.S. (2012). Successfully Improving Ocular Drug Delivery Using the Cationic Nanoemulsion, Novasorb. J. Drug Deliv..

[7] Liang H., Baudouin C., Faure M.O., Lambert G., Brignole-Baudouin F. (2009). Comparison of the ocular tolerability of a latanoprost cationic emulsion versus conventional formulations of prostaglandins: An in vivo toxicity assay. Mol. Vis..

[8] Daull P., Lallemand F., Garrigue J.S. (2014). Benefits of cetalkonium chloride cationic oil-in-water nanoemulsions for topical ophthalmic drug delivery. J. Pharm. Pharmacol..

[9] Tamilvanan S., Benita S. (2004). The potential of lipid emulsion for ocular delivery of lipophilic drugs. Eur. J. Pharm. Biopharm..

[10] Kauss T., Gaubert A., Tabaran L., Tonelli G., Phoeung T., Langlois M.H., White N., Cartwright A., Gomes M., Gaudin K. (2018). Development of rectal self-emulsifying suspension of a moisture-labile water-soluble drug. Int. J. Pharm..

[11] Rasoanirina B.N.V., Lassoued M.A., Kamoun A., Bahloul B., Miladi K., Sfar S. (2020). Voriconazole-loaded self-nanoemulsifying drug delivery system (SNEDDS) to improve transcorneal permeability. Pharm. Dev. Technol..

[12] Akula S., Gurram A.K., Devireddy S.R. (2014). Self-Microemulsifying Drug Delivery Systems: An Attractive Strategy for Enhanced Therapeutic Profile. Int. Sch. Res. Not..

[13] Tiwari R., Dubey V., Kesavan K. (2019). Ocular Self-Microemulsifying Drug Delivery System of Prednisolone Improves Therapeutic Effectiveness in the Treatment of Experimental Uveitis. Ocul. Immunol. Inflamm..

[14] Krzemińska K., Sznitowska M. (2023). Development of self-emulsifying oils for ophthalmic delivery of antibiotics instable in water. J. Drug Deliv. Sci. Tech..

[15] Wiącek A.E., Jurak M., Ładniak A., Przykaza K., Szafran K. (2020). Cyclosporine CsA-The Physicochemical Charac-terization of Liposomal and Colloidal Systems. Colloids Interfaces.

[16] Labbe A., Baudouin C., Ismail D., Amrane M., Garrigue J.S., Leonardi A., Figueiredo F.C., Van Setten G., Labetoulle M. (2017). Pan-European survey of the topical ocular use of cyclosporine A. J. Fr. Ophthalmol..

[17] De Campos A.M., Sánchez A., Alonso M.J. (2001). Chitosan nanoparticles: A new vehicle for the improvement of the delivery of drugs to the ocular surface. Application to cyclosporin A. Int. J. Pharm..

[18] Lallemand F., Felt-Baeyens O., Besseghir K., Behar-Cohen F., Gurny R. (2003). Cyclosporine A delivery to the eye: A pharmaceutical challenge. Eur. J. Pharm. Biopharm..

[19] Tang-Liu D.D.-S., Acheampong A. (2005). Ocular pharmacokinetics and safety of ciclosporin, a novel topical treatment for dry eye. Clin. Pharmacokinet..

[20] Utine C.A., Stern M., Akpek E.K. (2010). Clinical review: Topical ophthalmic use of cyclosporin A. Ocul. Immunol. Inflamm..

[21] Del Castillo J.M.B., Castillo A., Toledano N., Duran S., Del Aguila C., Otero M., Garcia-Sanchez J. (1995). Influence of topical Cyclosporine A and dissolvent on corneal epithelium permeability of fluorescein. Doc. Ophthalmol..

[22] Czajkowska-Kośnik A., Wolska E., Chorążewicz J., Sznitowska M. (2015). Comparison of cytotoxicity *in vitro* and irritation *in vivo* for aqueous and oily solutions of surfactants. Drug Dev. Ind. Pharm..

[23] Yenice I., Mocan M.C., Palaska E., Bochot A., Bilensoy E., Vural I., Irkec M., Atilla Hincal A. (2008). Hyaluronic acid coated poly-ε-caprolactone nanospheres deliver high concentrations of cyclosporine A into the cornea. Exp. Eye Res..

[24] Erawati T., Isadiartuti D., Anggalih B.D. (2023). The effect of polysorbate 20 and polysorbate 80 on the solubility of quercetin. J. Public Health Afr..

[25] Ravichandran V., Lee M., Nguyen Cao T.G., Shim M.S. (2021). Polysorbate- Based Drug Formulations for Brain-Targeted Drug Delivery and Anticancer Therapy. Appl. Sci..

[26] Wolska E., Sznitowska M., Chorążewicz J., Szerkus O., Radwańska A., Markuszewski M.J., Kaliszan R., Raczyńska K. (2018). Ocular irritation and cyclosporine A distribution in the eye tissues after administration of Solid Lipid Microparticles in the rabbit model. Eur. J. Pharm. Sci..

[27] Wilhelmus K.R. (2001). The Draize Eye Test. Surv. Ophthalmol..

[28] Lallemand F., Furrer P., Felt-Baeyens O., Gex-Fabry M., Dumont J.M., Besseghir K., Gurny R. (2005). A novel water-soluble cyclosporine A prodrug: Ocular tolerance and in vivo kinetics. Int. J. Pharm..

[29] Gan L., Gan Y., Zhu C., Zhang X., Zhu J. (2009). Novel microemulsion in situ electrolytetriggered gelling system for ophthalmic delivery of lipophilic cyclosporine A: In vitro and in vivo results. Int. J. Pharm..

[30] Shen J., Deng Y., Jin X., Ping Q., Su Z., Li L. (2010). Thiolated nanostructured lipid carriers as a potential ocular drug delivery system for cyclosporine A: Improving in vivo ocular distribution. Int. J. Pharm..

[31] Abdelkader H., Pierscionek B., Carew M., Wu Z., Alany R.G. (2015). Critical appraisal of alternative irritation models: Three decades of testing ophthalmic pharmaceuticals. Br. Med. Bull..

[32] Appendix B ICCVAM Summary Review Document: The Low Volume Eye Test. pp. 1–40.

[33] Ohno Y., Kaneko T., Inoue T., Morikawa Y., Yoshida T., Fujii A., Masuda M., Ohno T., Hayashi M., Momma J. (1999). Interlaboratory validation of the in vitro eye irritation tests for cosmetic ingredients. (1) Overview of the validation study and Draize scores for the evaluation of the tests. Toxicol. Vitr..

[34] Szerkus O., Wolska E., Struck-Lewicka W., Siluk D., Radwańska A., Wiczling P., Chorążewicz J., Sznitowska M., Markuszewski M.J., Kaliszan R. (2014). Development and validation of UHPLC method for the determination of cyclosporine A in biological samples. Biomed. Chromatogr..

